# Performance evaluation and multi-objective optimization of EDM parameters for Ti6Al4V using different tool electrodes

**DOI:** 10.1038/s41598-025-15756-5

**Published:** 2025-08-18

**Authors:** Manoj Jagdale, Masuk Abdullah, Nitin Ambhore, Atul Kulkarni, Rakesh Chaudhari, Jay Vora, József Menyhárt

**Affiliations:** 1 Department of Mechanical Engineering, Vishwakarma Institute of Information Technology, Savitribai Phule Pune University (SPPU), Pune, 411048 India; 2 Department of Vehicles Engineering, University of Debrecen, Ótemető strt. 2-4, Debrecen, 4028 Hungary; 3 Department of Mechanical Engineering, Vishwakarma Institute of Technology, Pune, 411037 India; 4 Department of Mechanical Engineering, School of Technology, Pandit Deendayal Energy University, Raisan, 382007 Gandhinagar India

**Keywords:** Ti6Al4V alloy, Copper tool, Brass tool, Graphite tool, Electrical discharge machining, Optimization, Engineering, Materials science

## Abstract

Ti6Al4V alloy is widely used in aerospace and biomedical applications due to its excellent mechanical and thermal properties, but its poor machinability makes it a difficult-to-cut material. Electrical Discharge Machining (EDM) offers an effective non-conventional machining approach for such materials, where tool electrode selection and process parameters critically influence performance. This study presents a comprehensive experimental investigation into the effect of three tool electrodes—graphite, copper, and brass—on the EDM performance of Ti6Al4V alloy. Key input parameters, including pulse-on time (T_on_), pulse-off time (T_off_), and current, were selected based on equipment limits and prior studies. Taguchi’s L9 orthogonal array was used for experimental design, and analysis of variance (ANOVA) was employed to determine the statistical significance of each factor. Output responses—material removal rate (MRR), tool wear rate (TWR), surface roughness (SR), and dimensional deviation (DD)—were measured and optimized using the Teaching–Learning-Based Optimization (TLBO) algorithm. Among the electrodes, graphite achieved the highest MRR (31.03 mm³/min), lowest TWR (0.4648 mm³/min), and minimal DD (101.76 μm), while brass produced the smoothest surface (SR = 3.19 μm). A collection of non-dominated responses was also found using Pareto optimal points. A minor adequate deviance was observed between the TLBO algorithm’s predicted and actual findings. Scanning electron microscopy (SEM) analysis was conducted to evaluate surface morphology. The qualitative SEM results confirmed fewer defects and better surface integrity for graphite electrodes. The findings validate TLBO as an effective tool for EDM process optimization and provide practical guidance for electrode selection in machining Ti6Al4V.

## Introduction

Titanium alloys are highly popular owing to their outstanding properties, such as higher strength, corrosion resistance, and favourable strength-to-weight ratio in various sectors^1,2^. Titanium alloys have been widely studied for the last few decades, and their importance has been recognized in various fields^3,4^. Ti6Al4V alloy is regarded as one of the most familiar titanium alloys, which can withstand high temperatures, making it suitable for applications in the aerospace industry, such as aircraft components, and automotive sectors like engine components^5,6^. Other applications of Ti6Al4V alloys include the biomedical sector, owing to their exceptional biocompatibility, aircraft, ships, nuclear energy plants, chemical vessels, recreational vehicles, and medical equipment^7,8^. Though the thermo-physical and mechanical properties of titanium alloys make them hard-to-cut materials with poor machinability^9–11^. The principal disadvantages of traditional methods of machining include low thermal conductivity, excessive chemical reactivity, and weak elastic modulus^12,13^. Non-conventional machining is employed to precisely shape complex and intricate geometries with high dimensional accuracy for difficult-to-cut materials^14,15^. The electric discharge machining (EDM) process, which removes the workpiece by generating sparks at the tool-work interface, is the most popular method for machining hard materials^16^. It becomes necessary to control EDM factors in order to get the desired machining performance^17,18^. Tool selection in the EDM method is important while machining difficult-to-cut materials such as Ti6Al4V alloy, as it affects the overall performance^19,20^. Researchers have studied the effect of different tools while controlling multiple factors on EDM of various difficult-to-cut materials.

Hamid et al.^21^ used the EDM process during the machining of NiTi alloy with graphite as the tool electrode. The input factors of Servo voltage (S_v_), discharge current (I_p_), pulse on time (T_on_), and pulse off time were studied on surface roughness (SR), tool wear rate (TWR), and material removal rate (MRR) using Taguchi’s design approach. The results revealed that current was having a major impact on output measures. The study reported by Esmail et al.^22^ has developed regression models with input factors of EDM and output measures while machining NiTi alloy using copper tools. Sana et al.^23^ employed Al6061 tool electrodes for the machining of aluminum 6061 work material of the EDM process, considering the input factors of S_v_, I_p_, T_on_, and Al_2_O_3_ powder concentration. The experiments were performed using the RSM approach, and output measures of TWR and accuracy index (AI) were analysed by using Grey relational analysis (GRA). In comparison with the conventional EDM process, the proposed empirical method has shown an enhancement of 5.11% and 48.29% for AI and TWR, respectively. The output measures of TWR and AI were significantly improved by 50.85% and 2.67%, respectively, by using the GRA optimization approach (Optimal EDM condition of I_p_: 5 A, T_on_: 3 µs, S_v_: 2 V, S_T_: and Al_2_O_3_ powder concentration: 1.5 g/100 ml). Farooq et al. 2023^24^ analysed the effect of four tool electrodes of copper, brass, aluminum, and graphite during the machining of Ti6Al4V ELI work material. The Adaptive Neuro-Fuzzy Inference System (ANFIS) approach was utilized to assess the performance of MRR, SR, and surface morphology. Electrode material and T_on_ have shown maximum contributions of 32.65% for MRR response and 32.15% for SR response, respectively. Copper electrodes have shown more surface defects like cracks, voids, and redeposited pieces of debris than graphite electrodes. Anshuman et al.^25^ used copper, graphite, and AlSiMg electrode prepared by using the SLS additive technique. The AlSiMg tool provided a superior surface texture compared to the copper and graphite electrodes. Ahmed et al.^26^ investigated the impact of tungsten carbide, brass, and copper tool electrodes on the EDM performance of titanium alloy. The brass tool electrode has shown maximum MRR, trailed by brass and tungsten carbide. Better surface quality has been observed with tungsten carbide tools as compared to brass and copper. Farooq et al. 2024^27^ used Aluminum and graphite electrodes along with other EDM input variables of T_on_, T_off_, non-ionic liquid type, and concentration for Ti6Al4V work material. The performance in terms of micro-machining errors was analysed using the desirability function. The optimized conditions recommend the use of a graphite electrode with non-ionic liquid S-60 with 25 g/L, T_on_ = 50 µs, and T_off_ = 50 µs. In addition to the above-mentioned literature, various attempts were made to enhance the machining performance by using several approaches, like Taguchi, RSM techniques with optimization techniques. Table 1 provides a comprehensive survey of the applied techniques to EDM.

**Table 1:** Comprehensive literature survey applied to EDM methods.

Authors, Years, and Ref.	Experimental conditions: Work-material; Tool electrode; Input variables.	Output measures; Optimization approach	Research findings
Sana et al. 2025^28^	Aluminum 6061; Non-treated and cryogenically treated brass tools; S_v_, T_on_, I_p_, and Al_2_O_3_ powder concentration.	MRR; SR, specific energy consumption (SEC); RSM with Non-dominated sorting genetic algorithm (NSGA-II).	The optimized process parameters with S_v_ = 2.18 V, T_on_ = 119.11 µs, I_p_ = 24.85 A,, and Al_2_O_3_ powder concentration = 1.05 g/100 ml of cryogenically treated brass tool has shown improvement in SR, SEC, and MRR by 27.45%, 46.60%, 64.82% in comparison with non-optimized parametric settings.
Hannan et al. 2024^29^	Stainless steel alloy SS310; Copper and brass electrodes; I_p_, spark gap, T_on_, duty cycle.	MRR, SR, TW, and EC Taguchi with composite desirability function.	Copper delivered higher MRR (2.67 mm³/min), lower SR (3.36 μm), lower TW (0.272 g), and lower EC (145.08 kJ) owing to its superior electrical/thermal properties as compared to the brass tool electrode.
Hurairah et al. 2024^30^	SS316; Copper, brass, and aluminum tool electrodes; Graphene-mixed dielectric versus standard dielectric.	Radial overcut (OC); Multi-objective Genetic Algorithm (MOGA) applied with Artificial Neural Network (ANN).	The obtained value greater than 0.99 for R² suggests the enhanced predictability of ANN. MOGA has shown that enhancement of 70.51% in OC response for copper tool when compared the results with the highest value of OC using the same copper tool electrode. Similarly, the brass and aluminum tools have shown improvement of 40.21% and 34.37%, respectively.
Tlija et al. 2024^31^	AISI D2 tool steel; Brass wire electrode; S_v_, T_on_, Material thickness, and wire tension (WT).	MRR and SR; Taguchi with composite desirability (dG)	The R² for both responses suggests a higher adequacy of the model. The optimal process parameter settings of S_v_ = 95 V, T_on_ = 4 µs, material thickness = 25.4 mm, and WT = 5 kgf were obtained with a dG value of 0.5614.
Sana et al. 2024^32^	Inconel 617; Non-treated and cryogenically treated brass tools; I_p_, S_v_, T_on_, powder concentration, surfactant concentration.	TWR and OC; MOGA applied with ANN.	The surfactant concentration was observed to have maximum impact on TWR (52.41%) and OC (72.67%). The optimized parameters have shown improvement of 47.05% and 85.00% for EWR and OC, respectively.
Hassan et al. 2024^33^	AISI D2 and DC53 hardened tool steels; Brass wire electrode; S_v_, T_on_, I_p_, and Material type.	SR; Taguchi with composite desirability (dG)	D2 steel achieved a better surface finish than DC53 on both flat and curved features. Under optimized conditions, SR improved by 66.03% on flat surfaces and by 60.09% on curved surfaces.
Sana et al. 2024^34^	Aluminum 6061; Non-treated and cryogenically treated brass tools; I_p_, S_v_, T_on_, powder concentration, surfactant concentration.	MRR, SR, SEC; NSGA-II with ANN.	ANN effectively established empirical relations among the input and output variables. NSGA-II optimized conditions have shown enhancement of 87.42%, 3.4%, and 0.7% in MRR, SR, and SEC, respectively. A reduction of 94.3% of CO_2_ emissions was attained by using deionized water.
Farooq et al. 2023^35^	Ti6Al4V ELI; Copper, brass, aluminum, and graphite; I_p_, T_on_, T_off_, powder concentration, electrode material, and polarity.	MRR and SR; Taguchi with GRA.	SiC powder in the dielectric reduced SR and produced finer surface texture. The simultaneous optimized parametric setting obtained through GRA has shown: Tool = Al electrode, I_p_ = 14 A, T_on_= 75 µs, T_off_ = 75 µs, and negative polarity.
Farooq et al. 2022^36^	Inconel 718; Brass wire electrode; S_v_, flushing pressure, nozzle diameter, nozzle-workpiece distance.	SR, spark gap, angular and radial deviations; Taguchi with GRA	The results obtained through GRA through optimized settings have resulted in 2.2 μm SR, 0.956% angular deviation, 3.49% radial deviation, and a spark gap of 0.109 mm. The modified flushing mechanism has shown enhancement of 1.92%, 8.24% and 29.11% for spark gap, angular, and radial deviations, respectively.
Rafaqat el al. 2022^37^	AISI D2 die steel; Copper tool with different relief angles; Tool designs with other constant factors of I_p_, T_on_, and T_off_.	MRR and TWR; Comparative experimental evaluation among designs.	The newly designed non-conventional electrode achieved roughly a 70% increase in MRR, a 45% reduction in TWR, and approximately 10% reduction in taper angle.
Chakraborty et al. 2023^38^	Ti6Al4V; Brass wire electrode; I_p_, T_on_, T_off_, and powder concentration.	SR, and corner in accuracy (CI); RSM with Teaching Learning-Based Optimization (TLBO).	The obtained findings have shown promising agreements with predicted findings from TLBO for both response measures, along with validation from ANOVA. The optimal settings attained through the TLBO technique have shown a minimum SR of 1.199 μm and CI of 12982.67 µm^2^. Improvement of 50.77% in SR and 23% in CI was observed in comparison with the conventional WEDM process.
Chakraborty et al. 2021^39^	Ti6Al4V; Brass wire electrode; I_p_, T_on_, T_off_, and B_4_C powder concentration.	SR, and corner profile error; GRA coupled with principal component analysis (GRA-PCA).	The optimized input setting for minimum SR of 1.315 μm and minimum CI of 11,623 µm^2^ was found as: T_on_ = 30 µs, T_off_ = 2 µs, I_p_ = 3 A, and B_4_C powder amount = 4 g/L. The GRA-PCA approach has shown a higher performance of 17.57% in comparison with the RSM approach.
Chakraborty et al. 2022^40^	Ti6Al4V; Brass wire electrode; I_p_, T_on_, T_off_, and powder concentration.	MRR and SR; Swarm optimization.	Powder-mixed deionized water (with surfactant) notably enhanced machining performance compared to pure water and kerosene. The optimized parametric settings of T_on_ = 30 µs, T_off_ = 7.24 µs, I_p_ = 2 A, and Al_2_O_3_ powder concentration = 4 g/L have shown a maximum MRR of 6.628 mm^3^/min and an average SR of 1.386 μm.
Sharma et al. 2024^41^	Ti-6Al-7Nb; Mo wire electrode; I_p_, T_on_, T_off_, and S_v_.	MRR and SR; Genetic Algorithm (GA) and TLBO method.	The optimized parametric settings obtained for simultaneous optimization using GA are: T_on_ 114 µs, T_off_ 60 µs, I_p_ 80 A, and S_v_ 80 V; and for TLBO are: T_on_ 114 µs, T_off_ 60 µs, I_p_ 140 A, and S_v_ 80 V. These optimized settings improved both MRR and SR over baseline experiments, demonstrating the effectiveness of ML-guided optimization on Ti-6Al-7Nb WEDM performance.
Harinath et al. 2025^42^	Tungsten carbide (WC); I_p_, T_off_, Spindle speed (SS), and wire feed (W_f_).	MRR and SR; TLBO method.	Among the several optimal solutions obtained by assigning the different weights to output responses in TLBO technique, the solution shown by equal weightage was optimal giving SR 2.45 μm, and MRR 5.2648 mm^3^/min at I_p_ = 4.4313 A, T_off_ = 1.297 µs, SS = 480.741 rpm, and W_f_ = 5.7502 m/min.

There are many different input and output parameters used in the EDM method. Controlling these factors is crucial, mainly when there is a conflict between the response variables^43,44^. During machining, many multi-objective optimization methods were used to achieve multiple objectives^45^. One such approach, Teaching Learning Based Optimization (TLBO), has an effective technique. TLBO is a meta-heuristic optimization technique that uses student-teacher interaction in a classroom. It functions as a technique based on human behavior. The TLBO algorithm identifies the global or near-optimal solutions for given objective functions. The TLBO algorithm was selected for this study due to its parameter-less structure, ease of implementation, and proven effectiveness in solving multi-objective optimization problems in manufacturing applications, including EDM^46–50^. Unlike other metaheuristic algorithms that require fine-tuning of control parameters (e.g., crossover and mutation rates in GA or inertia weights in PSO), TLBO eliminates this need, making it more robust and user-friendly for practical engineering problems.

As per the literature survey, for the machining of hard materials, the researchers largely used brass, copper, and graphite tool electrodes owing to their desired properties for the EDM process. The performance of the EDM process is largely dependent on the input variables and the combination of tool-work material. However, based on the reviewed literature and to the best of the author’s knowledge, a detailed comparative analysis of various tool electrodes combined with multi-response optimization has not been thoroughly explored for the Ti6Al4V alloy. Thus, the primary objective of this study is to experimentally investigate the influence of different tool electrodes (graphite, copper, and brass) on the EDM performance of Ti6Al4V alloy. Specific goals include:

1. Analyzing the effect of key input parameters (pulse-on time, pulse-off time, and current) on output responses such as MRR, TWR, SR, and DD;
2. Applying Taguchi’s L9 orthogonal array and ANOVA to identify significant process factors;
3. Optimizing multiple output responses using the TLBO algorithm; and.
4. Evaluating the surface integrity of machined samples through qualitative SEM analysis to assess the impact of electrode material on surface morphology.

This integrated approach aims to provide practical insights into electrode selection and process optimization for effective EDM machining of Ti6Al4V.

## Experimental plan

### Materials and methods

**Fig. 1:**
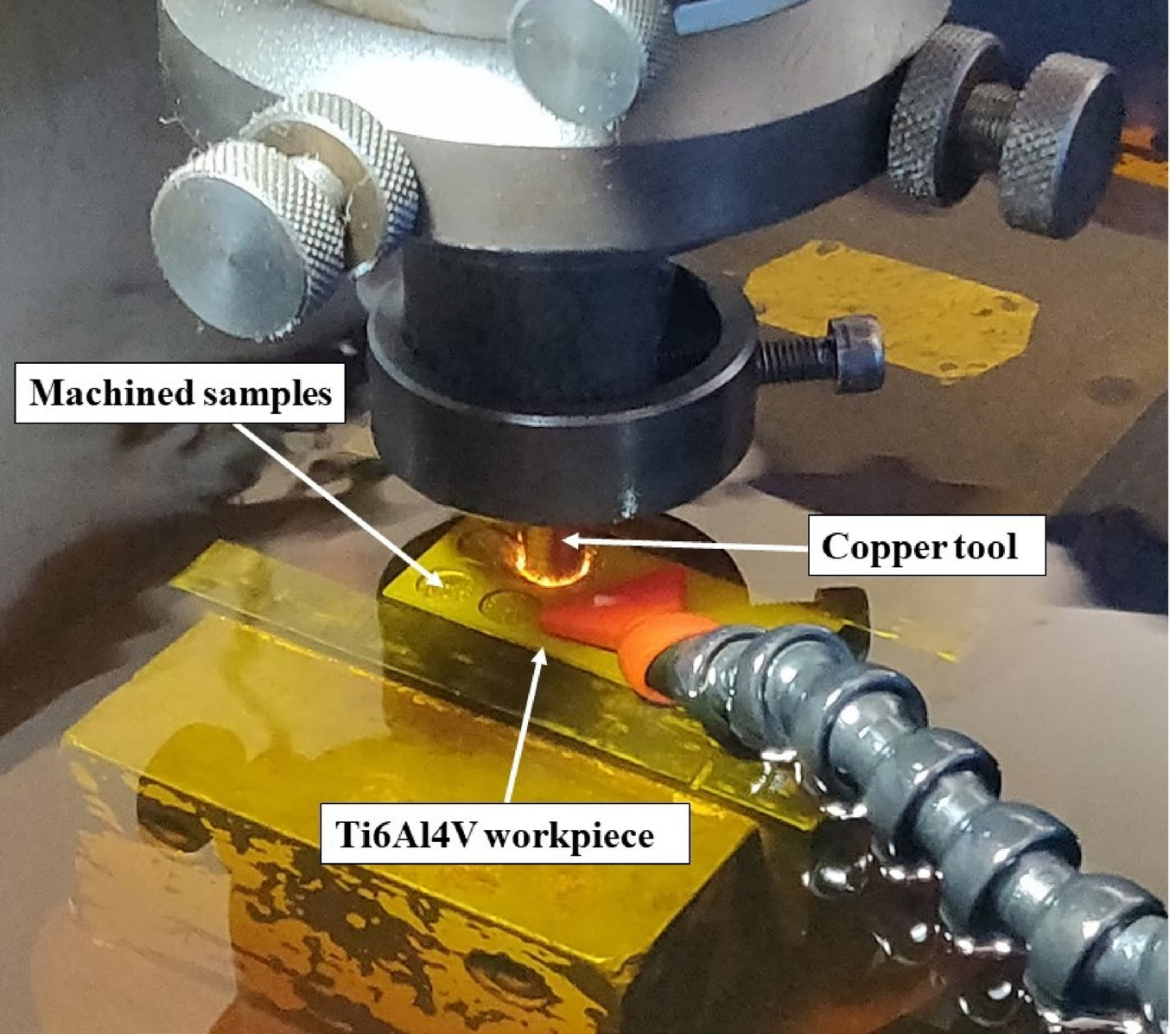
Experimental setup with Ti6Al4V work material and copper tool electrode.

In the present study, a spark erosion machine made by Sparkonix (Model S-50) has been employed to conduct the experimental trials. Figure 1 shows the experimental setup used in the present study along with the Ti6Al4V work material. The selected Ti6Al4V workpiece has 6% Al, 4% V, and the remaining percentage of titanium. Ti6Al4V alloy had a density of 4.45 g/cm^3^. Cylindrical cross-section tools of copper, brass, and graphite, having a diameter of 10 mm, were used as tool electrodes. Table 2 depicts the properties of selected tools and materials. Both the tool and work material were immersed in EDM oil during the machining. The input variables of T_on_, T_off,_ and I_p_ were selected with MRR, SR, TWR, and DD as performance measures.

**Table 2:** Characteristics of the selected tool materials.

Properties	Copper	Brass	Graphite
Melting point (°C)	1045	940	3650
Density (g/cm^3^)	8.96	8.73	1.85
Electrical conductivity (10^6^ S/m)	58.3	16	5.89
Thermal conductivity (W/mK)	380	160	120
Specific Heat Capacity (J/kgK)	384	380	707

Taguchi’s DOE approach was utilized to conduct the experimental runs. Taguchi’s design provides a robust experimental matrix with the minimum experimental runs^51^. The least number of trials also assesses the impact of input factors and saves cost and time^52^. The selection of the L9 array was based on the number of input parameters and their levels in this study. In the present study, three input factors at three levels were employed. The Taguchi L9 (3³) orthogonal array is well-suited for this configuration as it allows for an efficient and systematic exploration of the process space with a minimal number of experimental runs (9 instead of 27) while still capturing the main effects of each factor. This design significantly reduces experimental effort, cost, and time without compromising the ability to identify influential parameters and trends. The EDM factors with various levels are shown in Table 3. All the machined holes were given a depth of 2 mm by maintaining a spark gap of 0.01 mm. All the experiments were repeated three times, and the average value of the output response was considered to ensure reproducibility and accuracy of the results.

**Table 3:** Spark erosion parameters.

Input Variables	Values
T_on_ (µs)	4; 7; 10
T_off_ (µs)	3; 6; 9
Current (A)	10; 15; 20
Tool electrodes	Copper, Brass, Graphite

### Output responses

The material removal rate refers to the amount of rate of erosion of work material. It represents the machining capability for higher productivity and efficiency. The question (1) was used in the current study, which measures MRR in mm^3^/min. The ME204 analytical weighing balance, having a readability of 0.1 mg, was used to determine the work material weights.

1 $$\:\text{M}\text{R}\text{R}=\frac{\varDelta\:W\:\times\:1000}{\rho\:\:\times\:t}$$

where ΔW = difference in weight before and after machining, ρ = work material density in g/cm^3^, and t = machining time in minutes.

A similar equation was also employed to measure TWR in mm^3^/min.

Surface roughness (SR) indicates the smoothness of a machined area. Mitutoyo Surftest-410 was employed to measure the SR of the machined specimen by measuring at multiple locations, and the average value was taken for analysis. The cutoff value (λc) of 0.8 mm, along with 8 mm of evaluation length, was used for the measurement of SR as per the standard roughness measurement practices.

Another parameter that shows the accuracy of the process response is a dimensional deviation (DD), which was measured by using optical microscope for all machined surfaces. DD response depicts the difference between the actual diameter and the tool diameter after the machining operation. Finally, SEM was employed to reveal the machined surface topography for various tool electrodes.

### TLBO optimization

**Fig. 2:**
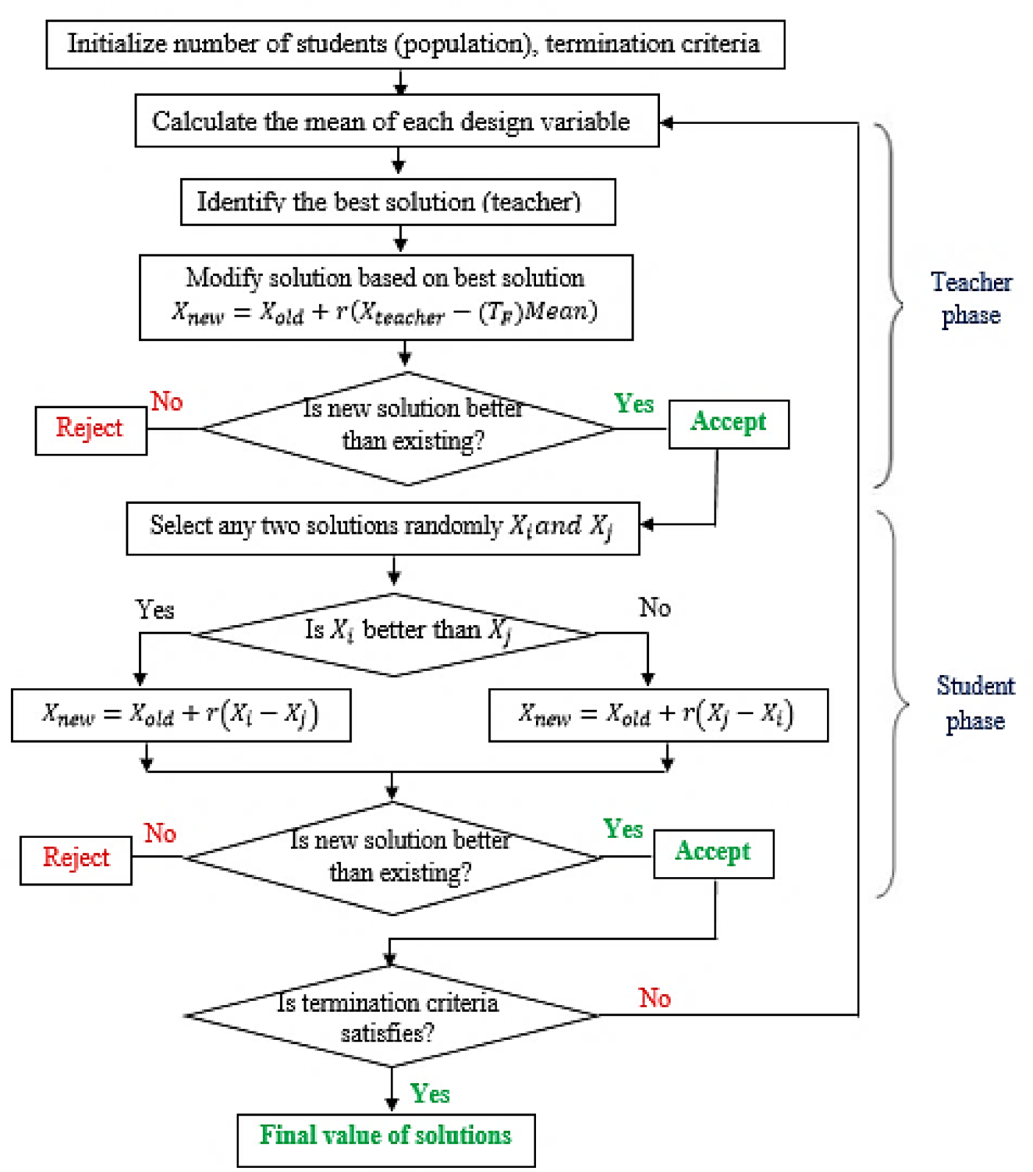
The procedure of the TLBO algorithm^53^.

In the present study, TLBO algorithm was employed to optimize the response variables. TLBO is a meta-heuristic optimization technique which uses student-teacher interaction in a classroom. It functions as a technique based on human behavior. The TLBO algorithm identifies the global or near-optimal solutions for given objective functions. A teacher’s impact on how students perform in a classroom is replicated by the TLBO algorithm. It operates in two main phases: the teacher phase and the learner phase. As a population-based method, it treats design variables as academic subjects being taught to students. In the teacher phase, the algorithm simulates an instructor delivering knowledge across various subjects (design parameters) to bring all students to a similar level of understanding. This is followed by the learner phase, where students improve through peer interactions and knowledge exchange. Just like academic performance is measured through assessments, the algorithm evaluates each student using fitness values. TLBO aims to optimize objective functions by enhancing these fitness values across the population. Following the teaching-learning process, a student’s level of skill matches an objective function’s fitness value. The teacher is the one with the best performance. Raising the class’s average performance from an initial level (M_1_) to the instructor’s level is the aim of the teacher phase. Still, it is not always possible to improve beyond M_1_ to a higher level (M_2_). If M_i_ is the class mean, as shown below, the teacher’s phase solution will be updated for both the old and new mean for each iteration. Figure 2 displays a detailed procedure for the implementation of the TLBO technique.

$$\begin{gathered} D{M_i}={\text{ }}{r_i}({M_{new}} - {T_F}{M_i}) \hfill \\ {T_F}={\text{ }}round{\text{ }}\left( 1 \right)\,+\,rand{\text{ }}\left( {0,{\text{ }}1} \right) \hfill \\ \end{gathered}$$

where *T*_*F*_ is the teaching factor, *r*_*i*_ is a random number between 0 and 1.

$${X_{new,i}}={\text{ }}{X_{old}}{,_i}+{\text{ }}D{M_i}_{{}}$$

In the students phase, taking two random learners X_k_ and X_k_ where j ≠ k.

$$\begin{gathered} If{\text{ }}f{\text{ }}\left( {{X_j}} \right)\,<\,f{\text{ }}\left( {{X_k}} \right), \hfill \\ {X_{new,j}}={\text{ }}{X_{old,j}}+{\text{ }}{r_j}({X_j} - {X_k}) \hfill \\ Otherwise \hfill \\ {X_{new,j}}={\text{ }}{X_{old,j}}+{\text{ }}{r_j}({X_k} - {X_j}) \hfill \\ \end{gathered}$$

## Results and discussions

The experimental values obtained for MRR, TWR, SR, and DD responses by following Taguchi’s approach have been represented in Table 4 for all the tool electrodes of copper, brass, and graphite.

**Table 4:** Results of measured responses following taguchi’s approach.

Run Order	T_on_	T_off_	I_*p*_	Copper Tool				Brass Tool				Graphite Tool			
				MRR	SR	TWR	DD	MRR	SR	TWR	DD	MRR	SR	TWR	DD
1	4	3	10	8.29	5.87	0.75	119.21	4.03	2.97	1.17	98.21	20.50	5.15	0.49	93.21
2	4	6	15	11.69	7.33	0.84	95.04	4.45	3.13	1.46	80.22	23.79	4.16	0.40	97.01
3	4	9	20	12.55	8.29	0.97	84.11	4.51	3.63	1.65	71.77	24.64	3.62	0.34	111.81
4	7	3	15	14.72	7.71	0.92	117.88	5.83	3.29	1.53	97.83	30.27	5.15	0.49	100.78
5	7	6	20	16.20	8.45	1.01	111.89	6.53	3.69	1.69	94.06	31.80	4.85	0.47	112.91
6	7	9	10	5.97	5.71	0.59	101.04	4.97	2.76	1.14	84.03	26.29	4.38	0.42	87.32
7	10	3	20	17.42	10.18	1.03	140.22	7.16	4.01	2.04	116.68	38.46	6.25	0.59	120.53
8	10	6	10	7.69	6.98	0.81	124.71	5.69	3.10	1.38	104.72	30.49	5.49	0.52	96.88
9	10	9	15	11.38	8.21	0.96	111.08	6.10	3.39	1.64	92.77	32.23	4.81	0.46	105.26

### Regression equations

Taguchi’s regression models were developed among the EDM factors and output measures for all tool electrodes by using Minitab v17 software^71^. The empirical relations for MRR, TWR, SR, and DD measures have been represented in Eqs. (2–5), respectively, for the copper tool electrode. Likewise, the empirical relations as shown in Eqs. (6–9) and (10–13) were also developed for brass and graphite tool electrodes, respectively.

*Regression Equations for Copper tool electrode*:

2 $$\:\text{M}\text{R}\:=1.62+0.220\cdot\:{\text{T}}_{\text{o}\text{n}\:}-0.585\cdot\:{\text{T}}_{\text{o}\text{f}\text{f}}+0.8076\cdot\:\text{C}\text{u}\text{r}\text{r}\text{e}\text{n}\text{t}$$

3 $$\:\text{T}\text{W}\text{R}\:=0.410+0.01351\cdot\:{\text{T}}_{\text{o}\text{n}\:}-0.00941\cdot\:{\text{T}}_{\text{o}\text{f}\text{f}}+0.02852\cdot\:\text{C}\text{u}\text{r}\text{r}\text{e}\text{n}\text{t}$$

4 $$\:\text{S}\text{R}\:=2.463+0.2157\cdot\:{\text{T}}_{\text{o}\text{n}\:}-0.0859\cdot\:{\text{T}}_{\text{o}\text{f}\text{f}}+0.2787\cdot\:\text{C}\text{u}\text{r}\text{r}\text{e}\text{n}\text{t}$$

5 $$\:\text{D}\text{D}\:=112.89+4.314\cdot\:{\text{T}}_{\text{o}\text{n}\:}-4.504\cdot\:{\text{T}}_{\text{o}\text{f}\text{f}}-0.291\cdot\:\text{C}\text{u}\text{r}\text{r}\text{e}\text{n}\text{t}$$

*Regression Equations for Brass tool electrode*:

6 $$\:\text{M}\text{R}\text{R}\:=1.879+0.3316\cdot\:{\text{T}}_{\text{o}\text{n}\:}-0.0798\cdot\:{\text{T}}_{\text{o}\text{f}\text{f}}+0.1168\cdot\:\text{C}\text{u}\text{r}\text{r}\text{e}\text{n}\text{t}$$

7 $$\:\text{T}\text{W}\text{R}\:=0.480+0.0433\cdot\:{\text{T}}_{\text{o}\text{n}\:}-0.0176\cdot\:{\text{T}}_{\text{o}\text{f}\text{f}}+0.0564\cdot\:\text{C}\text{u}\text{r}\text{r}\text{e}\text{n}\text{t}$$

8 $$\:\text{S}\text{R}\:=1.942+0.0425\cdot\:{\text{T}}_{\text{o}\text{n}\:}-0.0272\cdot\:{\text{T}}_{\text{o}\text{f}\text{f}}+0.08357\cdot\:\text{C}\text{u}\text{r}\text{r}\text{e}\text{n}\text{t}$$

9 $$\:\text{D}\text{D}\:=92.10+3.554\cdot\:{\text{T}}_{\text{o}\text{n}\:}-3.564\cdot\:{\text{T}}_{\text{o}\text{f}\text{f}}-0.148\cdot\:\text{C}\text{u}\text{r}\text{r}\text{e}\text{n}\text{t}$$

*Regression Equations for Graphite tool electrode*:

10 $$\:\text{M}\text{R}\text{R}\:=9.39+1.791\cdot\:{\text{T}}_{\text{o}\text{n}\:}-0.338\cdot\:{\text{T}}_{\text{o}\text{f}\text{f}}+0.5874\cdot\:\text{C}\text{u}\text{r}\text{r}\text{e}\text{n}\text{t}$$

11 $$\:\text{T}\text{W}\text{R}\:=0.4612+0.01917\cdot\:{\text{T}}_{\text{o}\text{n}\:}-0.01959\cdot\:{\text{T}}_{\text{o}\text{f}\text{f}}-0.00087\cdot\:\text{C}\text{u}\text{r}\text{r}\text{e}\text{n}\text{t}$$

12 $$\:\text{S}\text{R}\:=4.860+0.2011\cdot\:{\text{T}}_{\text{o}\text{n}\:}-0.2078\cdot\:{\text{T}}_{\text{o}\text{f}\text{f}}-0.0099\cdot\:\text{C}\text{u}\text{r}\text{r}\text{e}\text{n}\text{t}$$

13 $$\:\text{D}\text{D}\:=64.29+1.147\cdot\:{\text{T}}_{\text{o}\text{n}\:}-0.563\cdot\:{\text{T}}_{\text{o}\text{f}\text{f}}+2.261\cdot\:\text{C}\text{u}\text{r}\text{r}\text{e}\text{n}\text{t}$$

### ANOVA for responses

The outcomes of experimental trials were depicted in Table 4 for all three tool electrodes. The regression equations have been developed between the input and output factors for all tool electrodes. The statistical analysis of ANOVA was employed to assess the adequacy and reliability of the generated models. ANOVA was also used to determine the contribution of individual input factors to the output response using Minitab v17 software. The confidence interval of 95% was used for the analysis. The p-values below 0.05 are deemed statistically significant factors, and those P-values exceeding 0.05 were considered insignificant^54^. Table 5 shows the ANOVA results of MRR, TWR, SR, and DD responses for copper, brass, and graphite tools.

For copper tool electrodes, statistical analysis using ANOVA has revealed that the empirical regression model term of all the output responses was significant. This indicates that the overall regression model is statistically significant and reveals important interactions between the input and output factors. The lowest P-value for I_p_, and T_on_ has demostarted their significance for MRR response. The I_p_ was found to have a major impact on MRR, with 79.25% involvement, followed by T_off_ with 14.95% involvement. The least involvement with non-significance of T_on_ was observed for the MRR response. For SR response, T_off_ was a non-significant factor, while I_p_ and T_on_ were significant factors, giving major contributing factor of I_p_ (76.37%). For TWR response, with the involvement of 75.34%, the I_p_ was the only variable to have a substantial impact, while, other two input factors of T_on_ and T_off_ were non-significant. With a *p-*value of 0.001 and greater *f*-values, T_on_ and T_off_ were highly contributing factors for DD, with 45.06% and 49.13% contributions, respectively. The least involvement of 0.58% for I_p_ was found to be insignificant on DD response.

For brass tool electrodes, statistical analysis using ANOVA has revealed that the empirical regression model term of all the output responses was significant. This shows the adequacy of the developed regressions. The T_on_ was found to have maximum impact on MRR with 66.66% involvement, while I_p_ with maximum impact on both TWR and SR with 76.6% and 84.51% involvement, respectively, and T_off_ with maximum impact on DD with 47.53% involvement. With a *p-*value of 0.001 and greater *f*-values, T_on_ and T_off_ were highly contributing factors for DD, with 47.53% and 47.46% contributions, respectively. The least involvement of 0.22% for I_p_ was found to be insignificant on DD response.

For graphite tool electrodes, statistical analysis using ANOVA has revealed that the empirical regression model term of all the output responses was significant. This shows the adequacy of the developed regressions. The T_on_ was found to have maximum impact on MRR with 73.24% involvement, while T_off_ with maximum impact on both TWR and DD with 48.35% and 84.47% involvement, respectively, and I_p_ with maximum impact on SR with 49.03% involvement. The I_p_ contributed negligibly towards SR, showing a minor involvement of 0.31%. T_on_ and T_off_ have significantly affected the SR response. Lastly, current with the current highest contribution of 84.47% towards DD was observed.

**Table 5:** ANOVA results of response variables for copper, brass, and graphite tool electrodes.

Factors	Copper Tool				Brass Tool				Graphite Tool			
	SS	F	*P*	% contr.	SS	F	*P*	% contr.	SS	F	*P*	% contr.
MRR												
T_on_	0.00072	2.87	0.151	2.12	0.00164	56.07	0.001	66.66	0.04812	160.18	0	73.24
T_off_	0.00512	20.29	0.006	14.95	0.00009	3.25	0.132	3.65	0.00171	5.69	0.063	2.60
Current	0.02717	107.56	0.000	79.25	0.00056	19.34	0.007	22.76	0.01437	47.84	0.001	21.87
Error	0.00126			3.68	0.00014			5.68	0.00150			2.29
Total	0.03428				0.00246				0.06571			
TWR												
T_on_	0.00985	1.95	0.222	6.09	0.10113	18.1	0.008	16.24	0.01984	45.63	0.001	46.31
T_off_	0.00478	0.95	0.376	2.95	0.01668	2.99	0.145	2.68	0.02071	47.64	0.001	48.35
Current	0.12198	24.12	0.004	75.34	0.47706	85.41	0.000	76.60	0.00011	0.26	0.632	0.26
Error	0.02528			15.62	0.02793			4.48	0.00217			5.07
Total	0.16191				0.62279				0.04284			
SR												
T_on_	2.5129	18.14	0.008	16.48	0.09759	8.94	0.03	7.87	2.18286	48.27	0.001	45.91
T_off_	0.3989	2.88	0.151	2.62	0.03995	3.66	0.114	3.22	2.33127	51.55	0.001	49.03
Current	11.648	84.07	0.000	76.37	1.04768	95.99	0.000	84.50	0.01470	0.33	0.593	0.31
Error	0.6928			4.53	0.05457			4.40	0.22610			4.76
Total	15.252				1.23979				4.75492			
DD												
T_on_	1004.92	43.04	0.001	45.06	682.03	47.46	0.001	47.26	71	6.71	0.049	7.82
T_off_	1095.66	46.92	0.001	49.13	685.87	47.73	0.001	47.53	17.1	1.62	0.259	1.88
Current	12.73	0.55	0.493	0.58	3.3	0.23	0.652	0.22	767.04	72.54	0.000	84.47
Error	116.75			5.23	71.85			4.97	42.87			4.72
Total	2230.06				1443.05				908.02			

### Impact of EDM factors on output measures

The influence of EDM factors on output measures was examined by using main effect plots. The X-axis and Y-axis have been used to represent EDM factors and output measures, respectively.

#### Impact of T_on_ on output measures

**Fig. 3:**
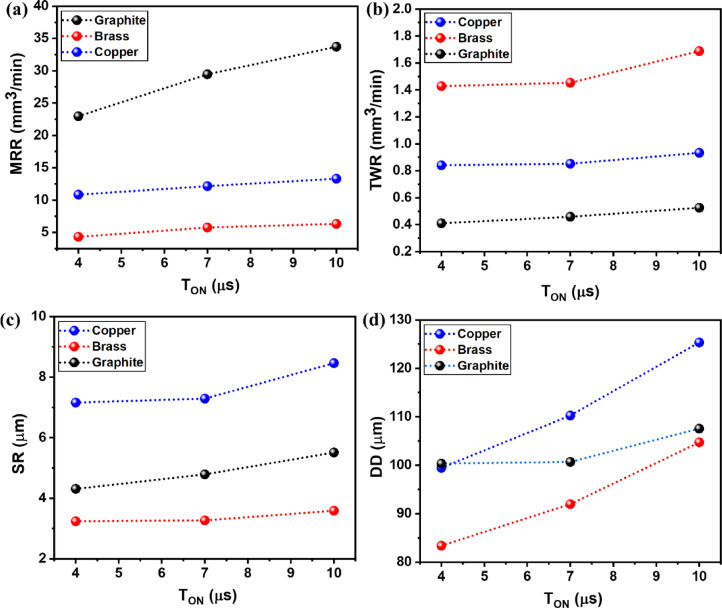
Effect of T_on_ on output measures of (**a**) MRR, (**b**) TWR, (**c**) SR, and (**d**) DD.

Figure 3a-d depicts the influence of the T_on_ variable on the output responses (MRR, TWR, SR, DD) for all three electrodes of brass, copper, and graphite. Figure 3a-d clearly shows the rise in T_on_ values has shown incremental trend for all response measures that too for all tool electrodes.

As depicted in Fig. 3a, MRR has increased with an increment in the T_on_ value. With the increase in T_on_, the number of active sparks rises, leading to greater thermal energy generation and enhanced melting and vaporization of the workpiece material. As a result, this leads to an increase in the MRR value. Markopoulos et al.^55^ and Vora et al.^56^ have observed similar trends during their study. For different tools, the largest MRR response was recorded with the use of graphite electrode as compared to brass and copper electrodes. The average MRR values for graphite were found to range from 22.97 mm³/min to 33.72 mm³/min, while for copper and brass, the mean MRR values ranged from 10.85 mm³/min to 13.31 mm³/min and 4.33 mm³/min to 6.32 mm³/min, respectively. The higher MRR observed with graphite is attributed to its relatively high melting point and specific heat capacity, which allow more heat to be transferred to the machining area instead of being absorbed by the tool itself^57^. The least melting point of brass amongst the three electrodes has resulted in lower MRR response compared to copper and graphite as it facilitated the erosion of material from the tool instead of workpiece^19^. Brass has significantly lower thermal and electrical conductivity than copper and graphite. This leads to less efficient energy transfer from the spark to the workpiece, resulting in weaker discharges and reduced erosion of the work material, hence lower MRR. For copper electride, the impact of T_on_ on MRR value was in-between the values of graphite and brass.

As per Fig. 3b, TWR increases gradually for all tools with the increased value of T_on_ owing to the higher discharge energies generated at the work-tool interface. Pursuant to this increased heat, the material is slowly eroded and melted off the tool material, whose magnitude depends on the tool electrode type. Thus, due to the lower melting point of brass compared to copper and graphite, it melts and deforms more easily and gives the highest wear rate^58^. Graphite has given the lowest TWR owing to its higher melting point than other tools. The obtained results agreed with the study by Singh et al.^59^. Average TWR values for graphite were found to in between 0.4105 mm^3^/min to 0.5255 mm^3^/min, while the average TWR values for copper and brass were 0.8406 mm^3^/min to 0.9337 mm^3^/min, and 1.4285 mm^3^/min to 1.6882 mm^3^/min respectively.

As depicted in Fig. 3c, SR has been increased with an increment in the T_on_ value. As discussed earlier, with the increased value of T_on_, the number of active sparks rises, leading to greater thermal energy generation and rise in temperature in machined zone^60^. The larger deeper craters generates the larger surface irregularities leading to higher SR values^61^. For copper tools, due to their high electrical conductivity compared to brass and graphite, a subsequent increase in sparks gave higher SR^57^. Average SR values for copper electrode were found to in between 7.16 μm and 8.46 μm, while the average SR values for brass and graphite were 3.24 μm to 3.59 μm and 4.31 μm to 5.51 μm respectively.

Due to the rise in T_on_, more sparks are generated, causing an immense rise in temperatures, thus removing more material. This increased removal causes DD in the machined parts. All tool materials showed an increasing slope for DD with the rise in T_on_. This is also evident from the study by Vora et al.^62^, where copper showed a growing trend of DD. The higher conductivity of copper tool electrodes has shown the most significant response values of DD as compared to brass and graphite. Average DD values for copper electrode were found to in between 99.45 μm and 125.33 μm, while the average DD values for brass and graphite were 83.4 μm to 104.72 μm and 100.33 μm to 107.55 μm respectively.

#### Impact of T_off_ on output measures

**Fig. 4:**
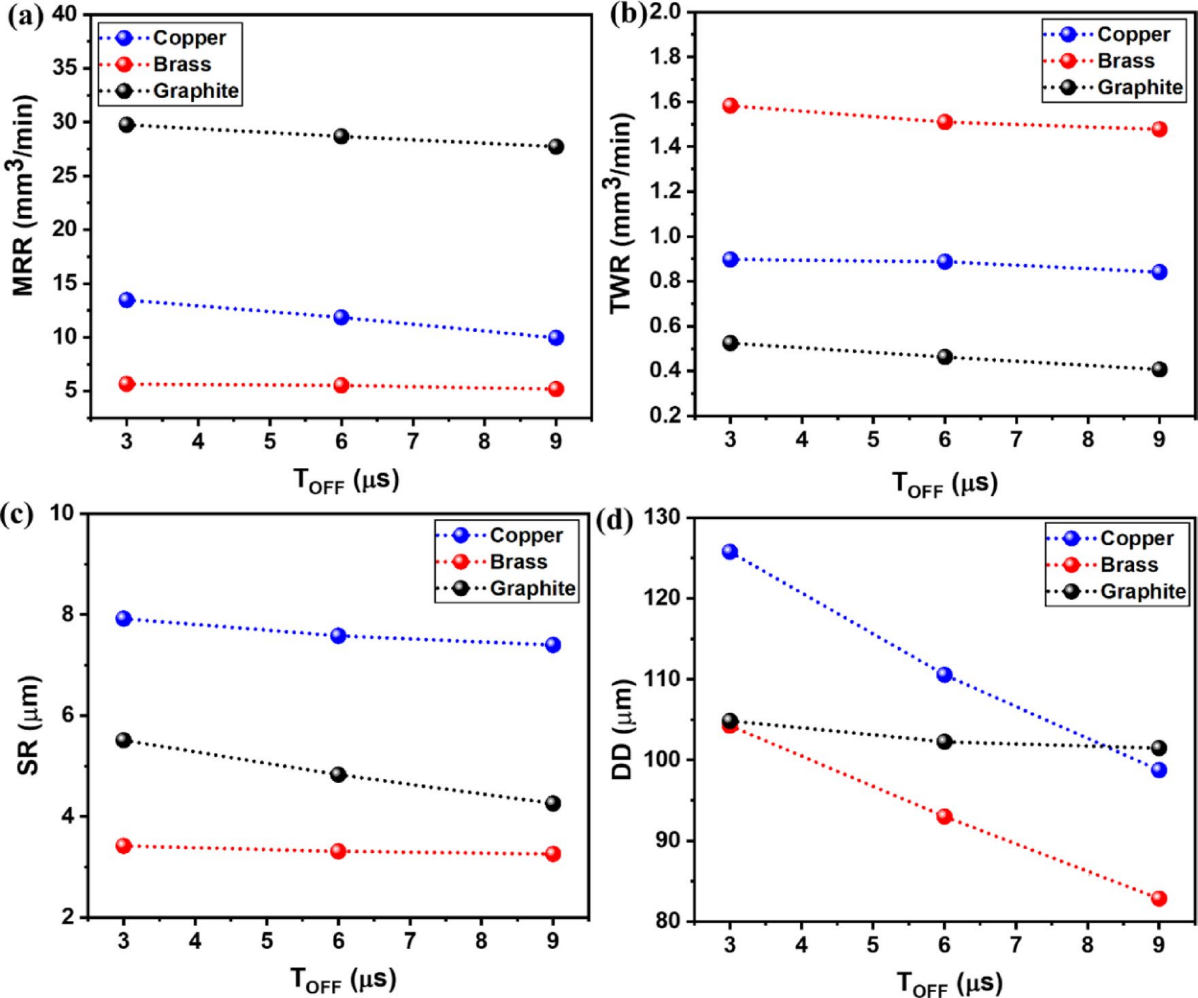
Effect of T_off_ on output measures of (**a**) MRR, (**b**) TWR, (**c**) SR, and (**d**) DD.

Figure 4a-d depicts the influence of the T_off_ variable on the output responses (MRR, TWR, SR, DD) for all three electrodes of brass, copper, and graphite. Figure 3a-d clearly shows that the rise in T_off_ values shows a decremental trend for all response measures that too for all tool electrodes.

As depicted in Fig. 4a, MRR has decreased with an increment in the T_off_ value. This was due to the fact that a larger T_off_ value intensifies the duration among the successive sparks, which in turn decreases the number of sparks generated per unit of time^63,64^. This generated lower thermal energy, and the rate of erosion lowers by showing lower MRR. A similar trend was observed in the case of all tool electrodes. For different tools, the largest MRR response was recorded with the use of graphite electrode as compared to brass and copper electrodes. The average MRR values for graphite were found to range from 29.75 mm^3^/min to 27.72 mm^3^/min, while for copper and brass, the mean MRR values ranged from 13.47 mm^3^/min to 9.96 mm^3^/min, and 5.67 mm^3^/min to 5.19 mm^3^/min, respectively. Graphite exhibited a higher MRR due to its relatively high melting point and specific heat capacity, which promote greater heat transfer to the machining zone instead of being retained by the tool. In contrast, brass showed a lower MRR compared to copper and graphite, primarily because its lower melting point leads to increased tool wear, resulting in more material being eroded from the tool rather than the workpiece.

TWR was found to have a declining trend with increased response values of T_off,_ as shown in Fig. 3c. The rise in T_off_ intensifies the duration among the successive sparks, which in turn decreases the number of sparks generated per unit of time. Thus, TWR decreases as it produces lower thermal energy at the work-tool interface. Brass has shown the highest TWR due to its lower melting point, while graphite has the lowest TWR due to its superior thermal properties compared to copper or brass^19^. Average TWR values for graphite were found to in between 0.5245 mm^3^/min to 0.4071 mm^3^/min, while the average TWR values for copper and brass were 0.898 mm^3^/min to 0.8415 mm^3^/min, and 1.5831 mm^3^/min to 1.4775 mm^3^/min, respectively.

DD has followed the same decreasing trend with an increase in T_off_. This leads to a reduction in material removal from the machined area. With the rise in T_off_ value, a larger amount of time gets lapsed, which in turn produces lower sparks. Thus, spark intensity also gets reduced, resulting in a lower temperature in the work-tool interference. This causes lower erosion of particles. Persuant to this, lower DD were produced due to the smaller amount of material elimination. The higher conductivity of copper tool electrodes has shown the most significant response values of DD compared to brass and graphite.

#### Impact of current on output measures

**Fig. 5:**
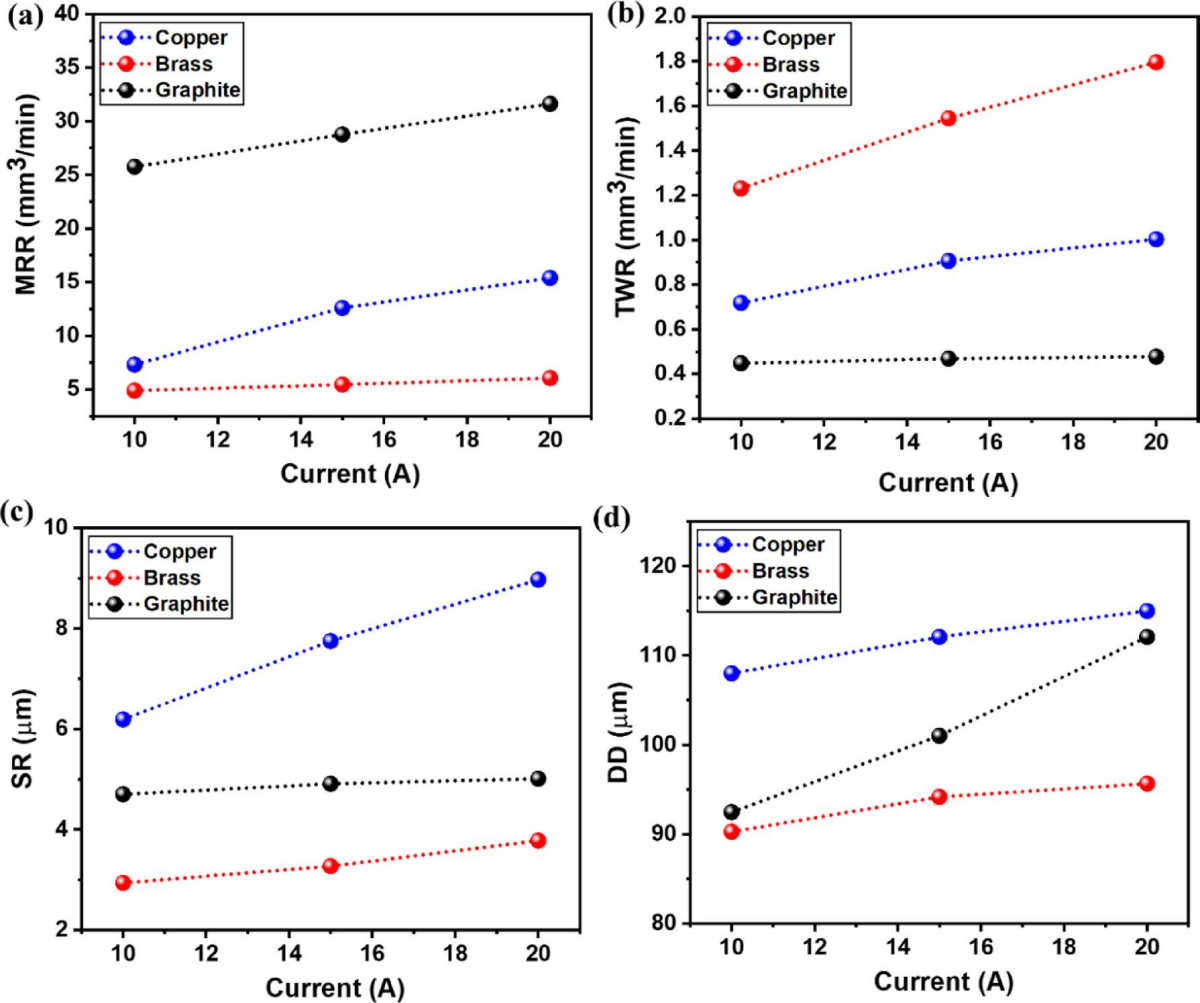
Effect of Current on output measures of (**a**) MRR, (**b**) TWR, (**c**) SR, (**d**) DD.

Figure 5a-d depicts the influence of the I_p_ variable on the output responses (MRR, TWR, SR, DD) for all three electrodes of brass, copper, and graphite. Figure 5a-d clearly shows the rise in I_p_ values has shown incremental trend for all response measures that too for all tool electrodes.

As depicted in Fig. 5, MRR has increased with an increment in the I_p_ value. The reason behind this is the increased discharge energy at the increased value of current. This, in turn, improves the sparking distribution, thereby improving the erosion rate as more materials get melted and vaporized^62^. As a result, this leads to an increase in the MRR value for all tool electrodes. For different tools, the largest MRR response was recorded with the use of graphite electrode as compared to brass and copper electrodes, owing to its relatively higher melting point and high specific heat capacity. The average MRR values for graphite were found to range from 25.76 mm^3^/min to 31.63 mm^3^/min, while for copper and brass, the mean MRR values ranged from 7.31 mm^3^/min to 15.39 mm^3^/min and 4.89 mm^3^/min to 6.06 mm^3^/min, respectively.

Figure 5b represents the mean effect of the current on TWR. Increased current value resulted in increased thermal energy of the spark zone, which erodes larger tool materials. Thus, all tool electrodes have shown increased TWR with an increase in current. Graphite has given the lowest TWR owing to its higher melting point than other tools^19^. Average TWR values for graphite were found to in between 0.4481 mm^3^/min to 0.4775 mm^3^/min, while the average TWR values for copper and brass were 0.718 mm^3^/min to 1.0031 mm^3^/min, and 1.2311 mm^3^/min to 1.7951 mm^3^/min, respectively.

As depicted in Fig. 5c, SR has been increased with an increment in the I_p_ value. With the increased value of I_p_, leading to greater thermal energy generation and a rise in temperature in the machined zone. The larger, deeper craters generate the larger surface irregularities, leading to higher SR values^65^. For copper tools, due to their high electrical conductivity compared to brass and graphite, a subsequent increase in sparks gave higher SR^66^. The obtained results were in agreement with the findings of Ahmed et al.^67^. Average SR values for copper electrode were found to in between 6.19 μm and 8.97 μm, while the average SR values for brass and graphite were 2.94 μm to 3.78 μm and 4.7 μm to 5.01 μm, respectively.

DD also shows an increasing trend for a rise in current values because of immense temperatures, leading to material erosion from the machined area’s edge and high deviation from the actual tool diameter^68^. The higher conductivity of copper tool electrodes has shown the largest response values of DD compared to brass and graphite. Average DD values for copper electrode were found to in between 108 μm and 114.98 μm, while the average DD values for brass and graphite were 90.27 μm to 95.65 μm, and 92.47 μm to 115.08 μm, respectively.

### Optimization

As per the main effect plots, it was apparent that each tool has different optimum levels of input factors for response measures of MRR, TWR, SR, and DD. The present study’s main objective is to maximize MRR while minimizing the SR, TWR, and DD. Such an objective gives a conflicting situation for the selection of input factors. Thus, multi-objective optimization technique of the TLBO algorithm was used for this purpose. The input factors were considered in lower and upper bounds as shown in Table 3.

#### Single-objective optimization

The main effect plots for selected input parameters show different levels for achieving the desired outcome. Single-response optimization has been employed to maximize MRR and minimize TWR, SR, and DD, and the results were depicted in Table 6 for copper, brass, and graphite tool electrodes. A conficting condition can be observed for all tool electrodes among the output responses with respect to the levels of input variables. Such a conflicting situation can be tackled by employing a simultaneous optimization approach.

**Table 6:** Single-objective optimization for copper.

Input Factors	T_on_	T_off_	Current	MRR	TWR	SR	DD
For Copper tool							
Maximum MRR	10	3	20	18.217	1.0872	9.94	136.69
Minimum TWR	4	9	10	5.311	0.6645	5.34	86.71
Minimum SR	4	9	10	5.311	0.6645	5.34	86.71
Minimum DD	4	9	20	13.387	0.9497	8.13	83.79
For Brass tool							
Maximum MRR	10	3	20	7.294	1.9882	3.96	113.98
Minimum TWR	4	9	10	3.663	1.0588	2.71	72.76
Minimum SR	4	9	10	3.663	1.0588	2.71	72.76
Minimum DD	4	9	20	4.831	1.6228	3.54	71.28
For Graphite tool							
Maximum MRR	10	3	20	38.034	0.5767	6.05	119.29
Minimum TWR	4	9	20	25.260	0.3441	3.59	109.03
Minimum SR	4	9	20	25.260	0.3441	3.59	109.03
Minimum DD	4	9	10	19.386	0.3528	3.69	86.42

#### Multi-objective optimization

Simultaneous optimization of all response variables was carried out using MOTLBO algorithm. For this, an equivalent weightage of 0.25 has been allocated to each response variable by considering the equal importance of all variables. Equation (14) shows the objective function for all the response measures.

14 $$\:Obj\:\left({v}_{4}\right)={w}_{1}\cdot\:\left(MRR\right)+{w}_{2}\cdot\:\left(TWR\right)+{w}_{3}\cdot\:\left(SR\right){\:+\:w}_{4}\cdot\:\left(DD\right)$$

This simultaneous optimization has yielded more favorable results for graphite tool electrodes. Table 7 depicted the findings of MOTLBO algorithm for all tool electrodes. The maximum MRR of 31.03 mm^3^/min, the least TWR of 0.4648 mm^3^/min, and the least DD of 101.76 μm has been obtained for graphite in comparison with brass and copper tools. The least value of SR (3.19 μm) was recorded in case of brass tool. To validate the findings from MOTLBO technique, verification trials were conducted and the results has shown an acceptable deviation of less than 4%. This suggests the adequacy of the generated models with TLBO method.

**Table 7:** Results of multi-objective optimization.

Type of tool	T_on_	T_off_	Current	MRR	TWR	SR	DD
Copper	6	5	20	16.167	1.0144	8.91	119.43
Brass	9	5	12	5.87	1.4585	3.19	104.49
Graphite	9	8	14	31.03	0.4648	4.87	101.76

By considering the suitability of graphite tool electrodes on response measures, 48 Pareto optimal points were generated in addition to the results of simultaneous optimizations. All these points provide unique optimal solutions. Table 8 shows these optimal solutions along with levels of input factors and outcomes of response measures. This gives a choice for users to appropriately pick the input factor conditions, considering their required values of response measures. Again, to validate these findings, a few verification trials were conducted, and the results have shown an acceptable deviation of less than 4%. This suggests the adequacy of the generated models with the TLBO method.

**Table 8:** Pareto optimal points for the graphite tool electrode.

Sr. No	Current	T_on_	T_off_	MRR	TWR	SR	DD
1	10	4	9	0.3232	0.3529	3.70	86.42
2	20	4	9	0.4211	0.3442	3.60	109.03
3	20	10	3	0.6340	0.5767	6.05	119.29
4	10	10	3	0.5361	0.5854	6.15	96.68
5	17	10	4	0.5990	0.5598	5.87	111.95
6	20	6	9	0.4808	0.3825	4.00	111.33
7	10	4	8	0.3289	0.3725	3.90	86.98
8	20	10	4	0.6284	0.5571	5.84	118.73
9	13	4	9	0.3526	0.3503	3.67	93.20
10	10	9	5	0.4950	0.5271	5.53	94.41
11	20	10	5	0.6228	0.5376	5.63	118.17
12	20	10	8	0.6059	0.4788	5.01	116.48
13	10	8	7	0.4539	0.4687	4.92	92.14
14	13	10	3	0.5655	0.5828	6.12	103.46
15	11	9	6	0.4992	0.5066	5.31	96.11
16	20	10	9	0.6002	0.4592	4.80	115.91
17	11	5	7	0.3741	0.4104	4.30	90.96
18	20	8	9	0.5405	0.4209	4.40	113.62
19	10	8	6	0.4595	0.4883	5.12	92.70
20	10	5	7	0.3643	0.4112	4.31	88.69
21	20	9	9	0.5704	0.4400	4.60	114.77
22	11	5	8	0.3685	0.3908	4.09	90.39
23	20	10	6	0.6171	0.5180	5.43	117.60
24	18	6	8	0.4669	0.4038	4.23	107.37
25	10	9	4	0.5006	0.5467	5.74	94.97
26	11	4	8	0.3387	0.3716	3.89	89.25
27	12	9	7	0.5033	0.4862	5.10	97.80
28	20	5	9	0.4510	0.3633	3.80	110.18
29	10	6	6	0.3998	0.4500	4.72	90.40
30	11	4	9	0.3330	0.3520	3.69	88.68
31	20	7	9	0.5107	0.4017	4.20	112.47
32	15	9	7	0.5327	0.4836	5.07	104.59
33	13	10	6	0.5486	0.5241	5.50	101.78
34	20	10	7	0.6115	0.4984	5.22	117.04
35	18	4	7	0.4128	0.3851	4.03	105.64
36	17	10	8	0.5765	0.4814	5.04	109.69
37	13	5	8	0.3881	0.3890	4.07	94.91
38	19	10	5	0.6130	0.5384	5.64	115.90
39	10	6	7	0.3942	0.4304	4.51	89.84
40	12	10	6	0.5388	0.5249	5.51	99.51
41	15	9	6	0.5383	0.5031	5.27	105.15
42	14	9	8	0.5173	0.4648	4.87	101.76
43	20	8	8	0.5462	0.4404	4.61	114.18
44	15	8	8	0.4972	0.4448	4.66	102.88
45	16	10	8	0.5667	0.4823	5.05	107.43
46	14	4	8	0.3680	0.3690	3.86	96.03
47	13	10	5	0.5542	0.5436	5.70	102.34
48	20	10	6	0.61712	0.51796	5.43	117.60

### Surface morphology of the machined surface

**Fig. 6:**
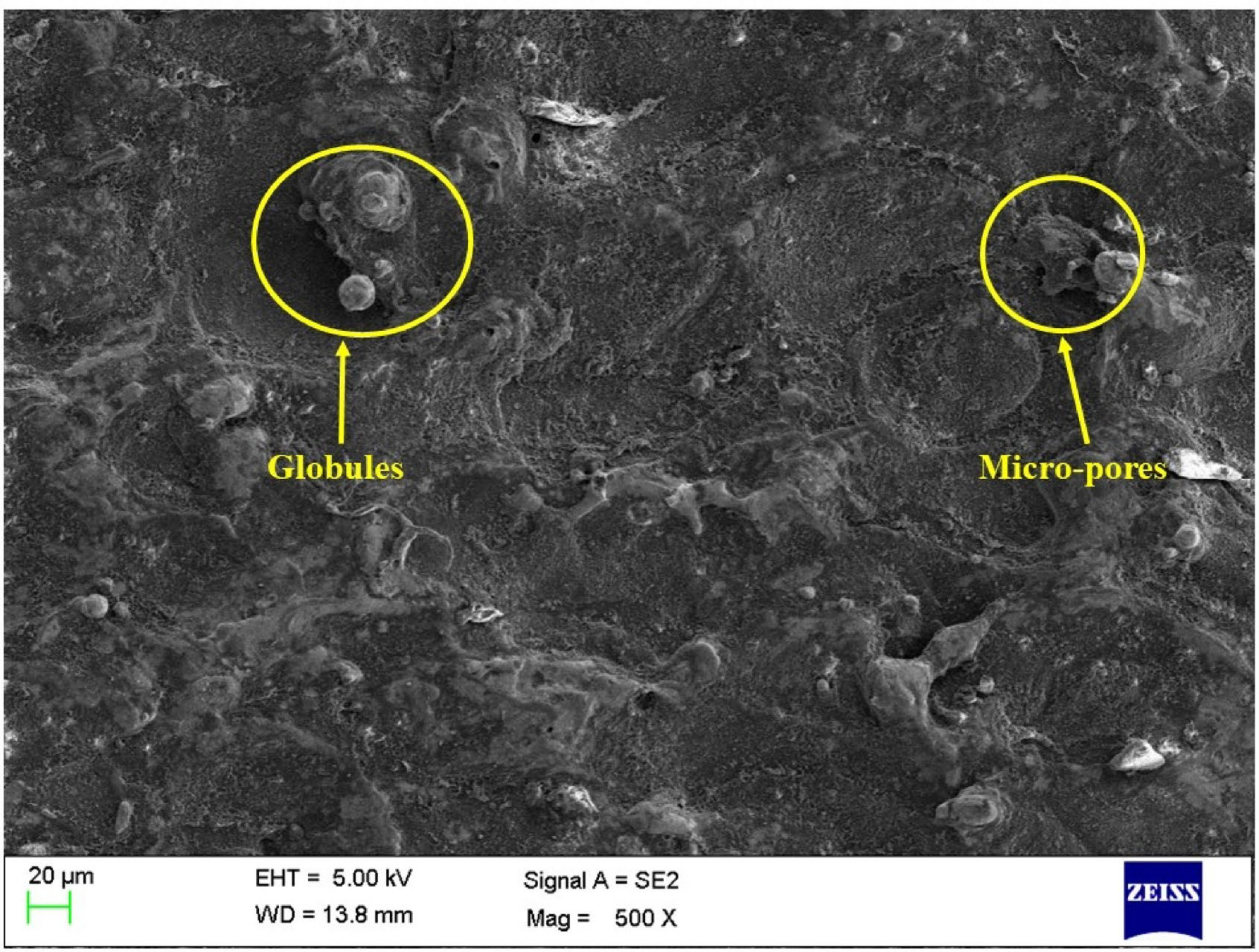
SEM of the machined surface by using a graphite tool electrode.

**Fig. 7:**
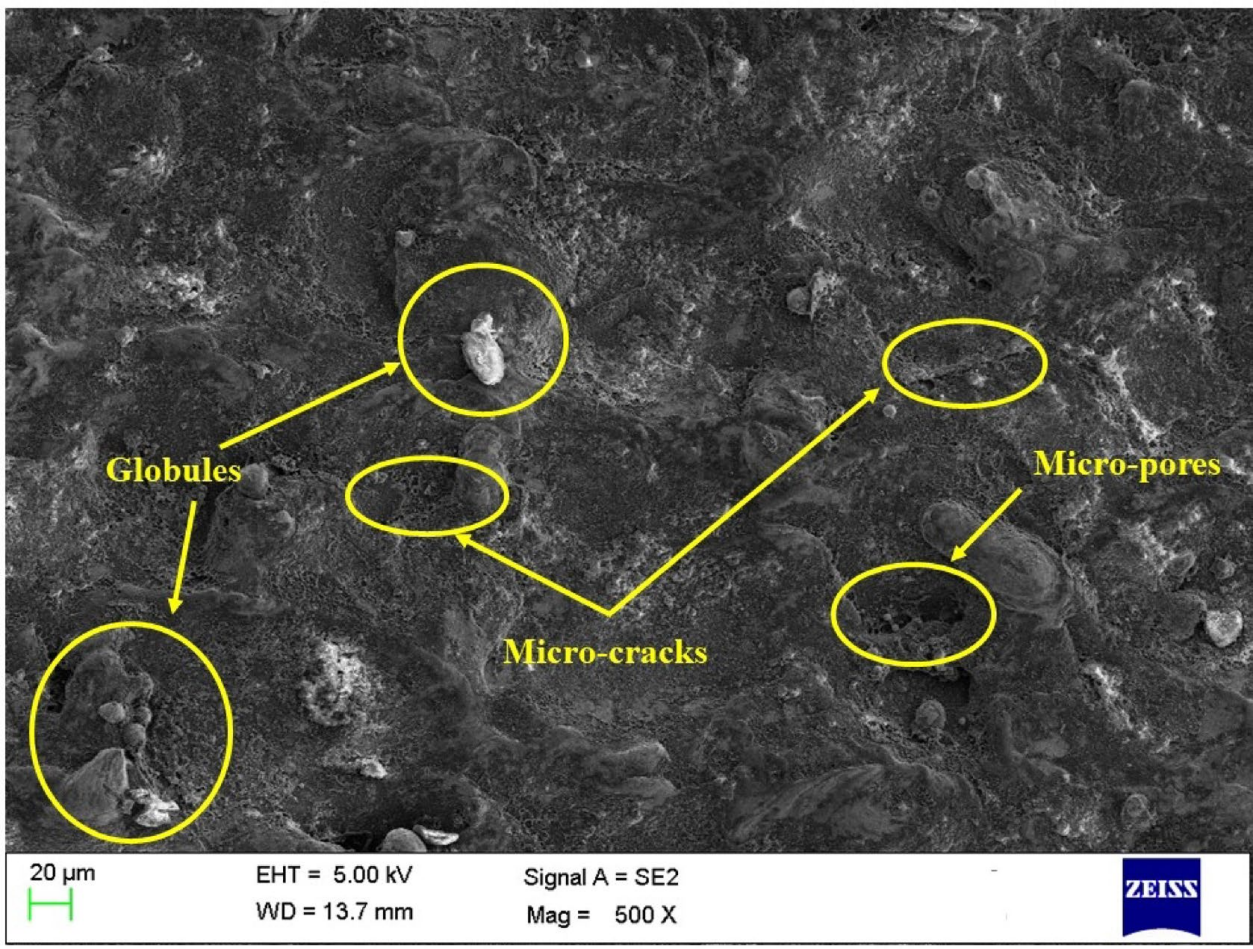
SEM of the machined surface by using a copper tool electrode.

**Fig. 8:**
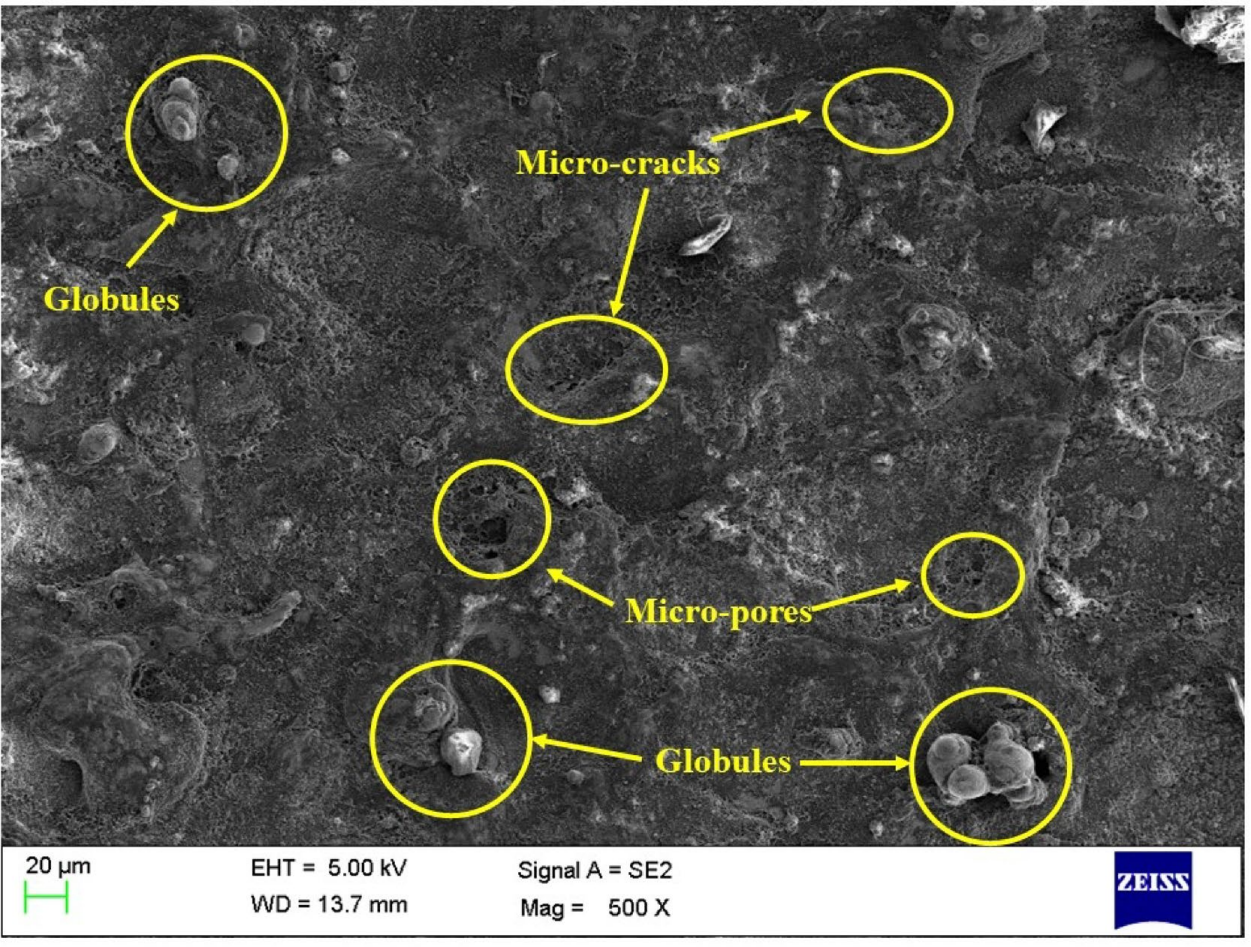
SEM of the machined surface by using a brass tool electrode.

Figures 6, 7 and 8 shows the SEM images of the machined surfaces obtained for graphite, copper, and brass electrodes, respectively. It is crucial to have input conditions in such a way that the machined surface will have minimal surface defects. The MOTLBO findings have shown the higher influence of graphite tool on output responses as compared to copper and brass tool electrodes. In addition to response values, surface defects also play a key role in the machine’s specimens. The surface machined with the graphite tool, as shown in Fig. 6, exhibited minimum microcracks, pores, and smaller globule sizes. This is attributed to graphite’s low thermal conductivity, which helps reduce thermal damage such as heat-affected zones, and thereby significantly minimizes surface defects^69^. Copper ranks at an intermediate level in performance, followed by brass. Due to its higher thermal conductivity compared to graphite, copper retains more heat during the EDM process. This leads to the formation of heat-affected zones and other thermal damage, thereby increasing surface defects. Additionally, copper’s lower melting point may cause material deposition on the machined surface during EDM, potentially resulting in a rougher surface finish^19^. Graphite and other electrode materials may provide a superior surface finish compared to copper, although copper can still be effective in certain EDM applications. Achieving the desired surface roughness with a copper tool may be possible through proper optimization of EDM process parameters. As shown in Fig. 8, the machined surface using the brass tool exhibited larger globules and more cracks, primarily due to the highest tool wear observed among all the electrodes^70^. This leads to increased melting of the tool material, followed by rapid cooling and adhesion to the machined surface, which in turn results in higher surface roughness.

## Conclusion

In the present study, experimental investigations were conducted using three different tool electrodes to evaluate the EDM performance of the Ti6Al4V alloy. The design variables of T_on_, T_off,_ and I_p_ were selected based on existing literature and the operational limits of the equipment. The output responses considered for analysis included MRR, TWR, SR, and DD. The obtained results have drawn the following conclusions:

- Regression models were developed using Minitab v17 software to establish relationships between input parameters and output responses for all three tool electrodes (graphite, copper, and brass).
- ANOVA results confirmed the statistical significance of the regression models across all tool materials and output measures, indicating strong correlations between machining parameters and performance outcomes. Increased T_off_ consistently resulted in reduced MRR and TWR, along with improved surface finish, across all electrode types.
- In terms of influential parameters: For MRR, current was the most significant factor for the copper tool, while T_on_ had the highest influence for graphite and brass tools. For TWR and SR, current was the dominant factor for copper and brass, whereas T_on_ was more significant for graphite. For DD, T_off_ was most significant for copper and brass, while current was dominant for graphite.
- The graphite electrode, due to its high melting point and thermal conductivity, demonstrated the highest MRR, followed by copper and then brass.
- In terms of tool wear and dimensional accuracy, graphite showed the lowest TWR and DD, while brass had the highest values, primarily influenced by the thermal properties of the electrode materials.
- The best surface finish (lowest SR of 3.19 μm) was achieved with the brass electrode, while copper produced the roughest surface. Graphite resulted in intermediate SR values, influenced by its thermal behavior and debris flushing efficiency.
- The TLBO algorithm was successfully applied to simultaneously optimize all four performance measures. The graphite tool yielded the best overall performance, achieving a maximum MRR of 31.03 mm³/min, a minimum TWR of 0.4648 mm³/min, and lowest DD of 101.76 μm.
- Pareto optimal solutions were generated to offer trade-off scenarios for multi-objective decision-making. Confirmatory experiments demonstrated less than 5% deviation from predicted values, validating the effectiveness of the TLBO approach.
- SEM analysis qualitatively revealed that graphite-produced surfaces exhibited fewer microcracks, smaller globules, and reduced porosity, confirming improved surface integrity compared to copper and brass tools.

Overall, this study offers practical insights into tool electrode selection and multi-response optimization for the EDM of Ti6Al4V alloy and supports the viability of TLBO as a robust method for process optimization.

## Data Availability

All data generated or analysed during this study are included in this published article.

## References

[1] Najafizadeh, M. et al. Classification and applications of titanium and its alloys: A review. *J. Alloy. Compd. Commun.* **3**, 100019 (2024).

[2] Tang, J. et al. Tribological performance and lubrication mechanism of polyacrylamide as a high-efficiency water-based lubricant additive for titanium alloys. *Sci. Rep.* **15** (1), 1–15 (2025). 40461603 10.1038/s41598-025-00737-5 PMC12134339

[3] Kishawy, H. A. et al. *Titanium and titanium alloys.* Machining difficult-to-cut materials: basic principles and challenges, : pp. 55–96. (2019).

[4] Yip, W. & To, S. Ductile and brittle transition behavior of titanium alloys in ultra-precision machining. *Sci. Rep.* **8** (1), 3934 (2018). 29500386 10.1038/s41598-018-22329-2 PMC5834465

[5] Jagdale, M. et al. Experimental investigation of process parameters in Wire-EDM of Ti-6Al-4 V. *Sci. Rep.* **15** (1), 5652 (2025). 39955453 10.1038/s41598-025-90486-2 PMC11830067

[6] Ma, L. et al. Combining laser biomimetic surface microtextured and PTFE solid lubricant for improved friction property of Ti6Al4V. *Sci. Rep.* **15** (1), 10457 (2025). 40140456 10.1038/s41598-025-94869-3 PMC11947283

[7] Safari-Gezaz, M., Parhizkar, M. & Asghari, E. Effect of Cobalt ions doping on morphology and electrochemical properties of hydroxyapatite coatings for biomedical applications. *Sci. Rep.* **15** (1), 149 (2025). 39747275 10.1038/s41598-024-84055-2 PMC11697187

[8] Farooq, M. U. et al. Application of non-ionic liquids-based modified dielectrics during electric discharge machining (EDM) of Ti6Al4V alloy to enhance machining efficiency and process optimization. *Sci. Rep.* **14** (1), 20797 (2024). 39242758 10.1038/s41598-024-71447-7 PMC11379698

[9] Kang, S. et al. Machining characteristics and process parameter optimization of Near-dry electrical discharge milling of titanium alloy. *Sci. Rep.* **15** (1), 8139 (2025). 40059190 10.1038/s41598-025-92830-y PMC11891323

[10] Gupta, V., Singh, B. & Mishra, R. Machining of titanium and titanium alloys by electric discharge machining process: a review. *Int. J. Mach. Mach. Mater.* **22** (2), 99–121 (2020).

[11] Bhargav, K. et al. Experimental investigation on machining characteristics of titanium processed using electrolyte sonicated µ-ECDM system. *Sci. Rep.* **12** (1), 15540 (2022). 36109662 10.1038/s41598-022-20001-4 PMC9478125

[12] Pramanik, A. et al. *Methods and variables in electrical discharge machining of titanium alloy–A review*. *Heliyon*, **6**(12). 10.1016/j.heliyon.2020.e05554 (2020). 10.1016/j.heliyon.2020.e05554 PMC7736727 33344787

[13] Jagadish et al. Optimization of process parameter for green die sinking electrical discharge machining: a novel hybrid decision-making approach. *Sci. Rep.* **15** (1), 13489 (2025). 40251287 10.1038/s41598-025-92713-2 PMC12008234

[14] Chaudhari, R. et al. Pareto optimization of WEDM process parameters for machining a NiTi shape memory alloy using a combined approach of RSM and heat transfer search algorithm. *Adv. Manuf.* **9** (1), 64–80 (2021).

[15] Muthuramalingam, T. et al. Multi-response optimization of EDM process parameters using assignments of weight method. *Int. J. Eng. Res. Technol.* **4** (16), 109–111 (2016).

[16] Al-Amin, M. et al. Powder mixed-EDM for potential biomedical applications: A critical review. *Mater. Manuf. Processes*. **35** (16), 1789–1811 (2020).

[17] Muthuramalingam, T. et al. *Multi-response optimization of WEDM process parameters of inconel 718 alloy using TGRA method*. in *International Conference on Engineering Research and Applications*. Springer. (2019).

[18] Chaudhari, R. et al. Multi-response optimization of WEDM process parameters for machining of superelastic nitinol shape-memory alloy using a heat-transfer search algorithm. *Materials* **12** (8), 1277 (2019). 31003478 10.3390/ma12081277 PMC6514827

[19] Harane, P. P., Wojciechowski, S. & Unune, D. R. Investigating the effect of different tool electrodes in electric discharge drilling of Waspaloy on process responses. *J. Mater. Res. Technol.* **20**, 2542–2557 (2022).

[20] Farooq, M. U. & Anwar, S. Investigations on the surface integrity of Ti6Al4V under modified dielectric (s)-based electric discharge machining using cryogenically treated electrodes. *Processes* **11** (3), 877 (2023).

[21] Sabouni, H. R. & Daneshmand, S. Investigation of the parameters of EDM process performed on smart NiTi alloy using graphite tools. *Life Sci. J.* **9** (4), 504–510 (2012).

[22] Abedi, E. et al. Analysis and modeling of electro discharge machining input parameters of nitinol shape memory alloy by de-ionized water and copper tools. *Int. J. Electrochem. Sci.* **9**, 2934–2943 (2014).

[23] Asad, M. et al. Producing micro impressions on Al6061 under alumina-mixed deionized water as dielectric during electric discharge machining. *J. Micromech. Microeng.* **35**(3), 035011 (2025).

[24] Farooq, M. U. et al. Electric discharge machining of Ti6Al4V ELI in biomedical industry: parametric analysis of surface functionalization and tribological characterization. *Materials* **16** (12), 4458 (2023). 37374641 10.3390/ma16124458 PMC10301990

[25] Sahu, A. K. et al. *Study on effect of tool electrodes on surface finish during electrical discharge machining of Nitinol*. in *IOP Conference Series: Materials Science and Engineering*. IOP Publishing. (2018).

[26] Khoshaim, A. B. et al. Influences of tool electrodes on machinability of titanium α-β alloy with ISO energy pulse generator in EDM process. *Alexandria Eng. J.* **63**, 465–474 (2023).

[27] Farooq, M. U., Anwar, S. & Hurairah, A. Reducing micro-machining errors during electric discharge machining of titanium alloy using nonionic liquids. *Mater. Manuf. Processes*. **39** (4), 449–464 (2024).

[28] Sana, M. et al. Sustainability metrics targeted optimization and electric discharge process modelling by neural networks. *Sci. Rep.* **15** (1), 3375 (2025). 39870639 10.1038/s41598-024-78883-5 PMC11772881

[29] Hannan, A. et al. Machining performance, economic and environmental analyses and multi-criteria optimization of electric discharge machining for SS310 alloy. *Sci. Rep.* **14** (1), 28930 (2024). 39572601 10.1038/s41598-024-79338-7 PMC11582806

[30] Hurairah, M. A. et al. Genetic algorithm-based optimization of artificial neural network of process parameters and characterization of machining errors in graphene mixed dielectric. *Arab. J. Sci. Eng.* **49** (11), 15649–15666 (2024).

[31] Tlija, M. et al. AISI D2 steel machining and manufacturing process optimization for tooling applications in biomedical industry. *AIP Adv.* **14**, 105–129. 10.1063/5.0217712 (2024).

[32] Sana, M. et al. Artificial neural networks-based modelling of effects of cryogenic electrode treatment, nano-powder, and surfactant-mixed dielectrics on wear performance and dimensional errors on Superalloy machining. *J. Brazilian Soc. Mech. Sci. Eng.* **46** (9), 539 (2024).

[33] Hassan, S. et al. Parametric analysis and multi-objective optimization for machining complex features on D2 and DC53 steels for tooling applications. *J. Mater. Eng. Polym.* **33**(21), 12109–12123 (2024).

[34] Sana, M. et al. Sustainable electric discharge machining using alumina-mixed deionized water as dielectric: process modelling by artificial neural networks underpinning net-zero from industry. *J. Clean. Prod.* **441**, 140926 (2024).

[35] Farooq, M. U. et al. Exploring wide-parametric range for tool electrode selection based on surface characterization and machining rate employing powder-mixed electric discharge machining process for Ti6Al4V ELI. *Int. J. Adv. Manuf. Technol.* **129** (5), 2823–2841 (2023).

[36] Farooq, M. U. et al. A novel Flushing mechanism to minimize roughness and dimensional errors during wire electric discharge machining of complex profiles on inconel 718. *Materials* **15** (20), 7330 (2022). 36295397 10.3390/ma15207330 PMC9607874

[37] Rafaqat, M. et al. Hole-making in D2-Grade steel tool by electric-discharge machining through non-conventional electrodes. *Processes* **10** (8), 1553 (2022).

[38] Chakraborty, S., Mitra, S. & Bose, D. Evaluation of response characteristics using sensitivity analysis and TLBO technique of powder mixed wire EDM of Ti6Al4V alloy. *CIRP J. Manufact. Sci. Technol.* **47**, 260–272 (2023).

[39] Chakraborty, S., Mitra, S. & Bose, D. *An investigation on dimensional accuracy and surface topography in powder mixed WEDM using RSM and GRA-PCA.* Materials Today: Proceedings, 44: pp. 1524–1530. (2021).

[40] Chakraborty, S., Mitra, S. & Bose, D. *Performance characterization of powder mixed wire electrical discharge machining technique for processing of Ti6Al4V alloy.* Proceedings of the Institution of Mechanical Engineers, Part E: Journal of Process Mechanical Engineering, 236(4): pp. 1283–1295. (2022).

[41] Sharma, V., Misra, J. P. & Singhal, S. Machine learning algorithms based advanced optimization of wire-EDM parameters: an experimental investigation into titanium alloy. *Int. J. Interact. Des. Manuf. (IJIDeM)*. **18** (5), 2855–2868 (2024).

[42] Harinath, M. & Parthiban, M. Experimental investigation on WEDG machining parameters for tungsten carbide. *Mater. Manuf. Processes*. **40** (1), 95–103 (2025).

[43] Kumar, L., Kumar, K. & Chhabra, D. Experimental investigations of electrical discharge micro-drilling for Mg-alloy and multi-response optimization using MOGA-ANN. *CIRP J. Manuf. Sci. Technol.* **38**, 774–786 (2022).

[44] Prakash, C. et al. Multi-objective particle swarm optimization of EDM parameters to deposit HA-coating on biodegradable Mg-alloy. *Vacuum* **158**, 180–190 (2018).

[45] Al-Amin, M. et al. Analysis of hybrid HA/CNT suspended-EDM process and multiple-objectives optimization to improve machining responses of 316L steel. *J. Mater. Res. Technol.* **15**, 2557–2574 (2021).

[46] Chaudhari, R. et al. Parametric optimization and influence of near-dry WEDM variables on nitinol shape memory alloy. *Micromachines* **13** (7), 1026 (2022). 35888844 10.3390/mi13071026 PMC9320167

[47] Chaudhari, R. et al. *Optimization of Parameters of Spark Erosion Based Processes, in Spark Erosion Machining*p. 190–216 (CRC, 2020).

[48] Singh, H. et al. An integrative TLBO-driven hybrid grey Wolf optimizer for the efficient resolution of multi-dimensional, nonlinear engineering problems. *Sci. Rep.* **15** (1), 11205 (2025). 40169707 10.1038/s41598-025-89458-3 PMC11962168

[49] Singh, A. et al. Optimized PID controller and model order reduction of reheated turbine for load frequency control using teaching learning-based optimization. *Sci. Rep.* **15** (1), 3759 (2025). 39885222 10.1038/s41598-025-87866-z PMC11782507

[50] Srinivasan, V. et al. *Comparative study on EDM parameter optimization for adsorbed Si3N4–TiN using TOPSIS and GRA coupled with TLBO algorithm.* Adsorption Science & Technology, 2022: p. 4112448. (2022).

[51] Moayyedian, M., Qazani, M. R. C. & Pourmostaghimi, V. Optimized injection-molding process for thin-walled polypropylene part using genetic programming and interior point solver. *Int. J. Adv. Manuf. Technol.* **124** (1–2), 297–313 (2023).

[52] Huu Phan, N. & Muthuramalingam, T. Multi criteria decision making of vibration assisted EDM process parameters on machining silicon steel using Taguchi-DEAR methodology. *Silicon* **13** (6), 1879–1885 (2021).

[53] Chaudhari, R. et al. *Multi-response optimization of Al2O3 nanopowder-mixed wire electrical discharge machining process parameters of nitinol shape memory alloy.* Materials, 15(6): p. 2018. (2022). 10.3390/ma15062018 PMC8950695 35329469

[54] Huu, P. N. et al. Optimizing surface quality in PMEDM using SiC powder material by combined solution response surface methodology–Adaptive neuro fuzzy inference system. *J. Mech. Behav. Mater.* **34** (1), 20250051 (2025).

[55] Markopoulos, A. P., Papazoglou, E. L. & Karmiris-Obratański, P. *Experimental study on the influence of machining conditions on the quality of electrical discharge machined surfaces of aluminum alloy Al5052.* Machines, 8(1): p. 12. (2020).

[56] Vora, J. et al. Multi-response optimization and influence of expanded graphite on performance of WEDM process of Ti6Al4V. *J. Manuf. Mater. Process.* **7** (3), 111 (2023).

[57] Kuppan, P., Narayanan, S. & Rajadurai, A. Effect of process parameters on material removal rate and surface roughness in electric discharge drilling of inconel 718 using graphite electrode. *Int. J. Manuf. Technol. Manage.* **23** (3–4), 214–233 (2011).

[58] Tsai, Y. Y. & Masuzawa, T. An index to evaluate the wear resistance of the electrode in micro-EDM. *J. Mater. Process. Technol.* **149** (1–3), 304–309 (2004).

[59] Singh, S., Maheshwari, S. & Pandey, P. Some investigations into the electric discharge machining of hardened tool steel using different electrode materials. *J. Mater. Process. Technol.* **149** (1–3), 272–277 (2004).

[60] Chaudhari, R. et al. Surface analysis of wire-electrical-discharge-machining-processed shape-memory alloys. *Materials* **13** (3), 530 (2020). 31979023 10.3390/ma13030530 PMC7040585

[61] Nguyen, H. P., Ngo, N. V. & Nguyen, Q. T. Optimizing process parameters in edm using low frequency vibration for material removal rate and surface roughness. *J. King Saud University-Engineering Sci.* **33** (4), 284–291 (2021).

[62] Vora, J. et al. Machining parameter optimization and experimental investigations of nano-graphene mixed electrical discharge machining of nitinol shape memory alloy. *J. Mater. Res. Technol.* **19**, 653–668 (2022).

[63] Al-Amin, M. et al. Multi-objective optimization of process variables for MWCNT-added electro-discharge machining of 316L steel. *Int. J. Adv. Manuf. Technol.* **115**(1), 179–198 (2021).

[64] Chaudhari, R. et al. Effect of WEDM process parameters on surface morphology of nitinol shape memory alloy. *Materials* **13** (21), 4943 (2020). 33153190 10.3390/ma13214943 PMC7663334

[65] Goyal, A. et al. Experimental investigation for minimizing circularity and surface roughness under nano graphene mixed dielectric EDM exercising fuzzy-ANFIS approach. *Int. J. Interact. Des. Manuf.* **16**(3), 1135–1154 (2022).

[66] Haron, C. C. et al. Copper and graphite electrodes performance in electrical-discharge machining of XW42 tool steel. *J. Mater. Process. Technol.* **201** (1–3), 570–573 (2008).

[67] Ahmed, N. et al. Machinability of titanium alloy through electric discharge machining. *Mater. Manuf. Processes*. **34** (1), 93–102 (2019).

[68] Parsana, S. et al. Machining parameter optimization for EDM machining of Mg-RE-Zn-Zr alloy using multi-objective passing vehicle search algorithm. *Archives Civil Mech. Eng.* **18** (3), 799–817 (2018).

[69] Moudood, M. et al. Investigation of the machininabilty of Non-Conductive ZrO2 with different tool electrodes in EDM. *Int. J. Automot. Mech. Eng.* **10**, 1866–1876 (2014).

[70] Sahu, A. K. & Mahapatra, S. S. Comparison of performance of different tool electrodes during electrical discharge machining, Vol. 26 (2019).

[71] Minitab *Statistical Software*, available: https://www.minitab.com/en-us/products/minitab/ (accessed: 2025-07-03).

